# Salvage Total Hip Arthroplasty After Cephalomedullary Nail Failure Due to Nonunion in a Pertrochanteric Fracture: A 10-Year Follow-Up

**DOI:** 10.7759/cureus.98973

**Published:** 2025-12-11

**Authors:** Hassan Zmerly, Rebecca Rauch, Manuela Moscato, Ibrahim Akkawi, Francesco Pegreffi

**Affiliations:** 1 1st Unit of Orthopedics, Villa Erbosa, Bologna, ITA; 2 Faculty of Medicine and Surgery, Link Campus University, Rome, ITA; 3 Department of Orthopedics and Traumatology, ASST (Azienda Socio-Sanitaria Territoriale) Gaetano Pini Hospital, University of Milano, Milano, ITA; 4 Department of Orthopedics, San Pier Damiano Hospital, GVM Care and Research, Faenza, ITA; 5 5th Unit of Orthopedics, Villa Erbosa, Bologna, ITA; 6 Department of Medicine and Surgery, University of Enna “Kore”, Enna, ITA; 7 Unit of Recovery and Functional Rehabilitation, Umberto I Hospital, Enna, ITA

**Keywords:** blood supply management, gamma nail, hip total arthroplasty, intramedullary nail, nail breakage, nonunion, perthocaneric fracture

## Abstract

Cephalomedullary nailing is a widely used treatment for pertrochanteric fractures because of its minimally invasive nature, low complication rate, and biomechanical advantages. However, nonunion remains a rare but severe complication that can lead to implant failure and eventual nail breakage.

We report the case of an 81-year-old patient who presented with implant failure following fixation of a pertrochanteric fracture with a Gamma cephalomedullary nail. Postoperatively, the patient developed nonunion, and nail breakage occurred 13 months after the initial surgery. Management was further complicated by the patient’s refusal of an allogeneic blood transfusion. The broken nail was removed, and the patient successfully underwent a cementless total hip arthroplasty. At the 10-year follow-up, a clinical examination confirmed excellent functional recovery, and plain radiographs showed stable implant fixation with no evidence of loosening or other complications.

This case highlights that conversion to total hip arthroplasty following nonunion-related Gamma nail breakage is a viable and effective option in elderly patients, providing long-term pain relief and stable implant survival, even in the absence of allogeneic blood transfusion.

## Introduction

Pertrochanteric fractures are among the most common injuries in elderly patients and are typically managed with intramedullary fixation. Gamma cephalomedullary nails are widely used because they provide high biomechanical stability and enable early mobilization and weight-bearing [1,2]. Although intramedullary nailing is associated with high union rates, one of its most concerning complications is nonunion, which can ultimately result in implant failure and the dreaded event of hardware breakage [3]. Risk factors for nonunion pertrochanteric fracture include inadequate initial fracture reduction or excessive fracture displacement, suboptimal implant positioning (e.g., high or lateral placement of the cephalic screw), poor bone quality (osteoporosis), and early, excessive weight-bearing [1-5].

Nail breakage usually occurs when the mechanical stress on the implant exceeds the load-bearing capacity of the healing bone. The factors contributing to implant failure include suboptimal fracture reduction, inadequate fixation, poor callus formation, and excessive mechanical loading during rehabilitation [4,5].

When implant failure occurs, several revision strategies may be considered, such as exchange nailing, plate fixation, or conversion to total hip arthroplasty (THA), depending on the patient’s general condition, age, and bone quality. Exchange nailing and plate fixation are generally preferred in younger patients, as these techniques help preserve native bone stock and support long-term functional outcomes; however, in elderly patients with concomitant hip osteoarthritis, conversion to THA often represents the most appropriate option, as it provides reliable pain relief and superior functional recovery [6-10].

We report a case of intramedullary nail breakage secondary to nonunion of a pertrochanteric fracture that required revision surgery with conversion to THA. The management was further complicated by the patient’s refusal of allogeneic blood transfusion, which can pose a life-threatening risk in the event of excessive blood loss, necessitating the implementation of meticulous perioperative blood conservation strategies.

## Case presentation

Patient history and initial treatment

An 81-year-old male patient with a medical history of osteoporosis and mild cognitive impairment sustained a low-energy pertrochanteric femoral fracture following an accidental fall at home. The fracture was treated by intramedullary fixation using a Gamma cephalomedullary nail, consisting of a proximal femoral nail, cephalic lag screw, and distal locking screw. Postoperatively, partial weight-bearing with the assistance of a walker was advised. Immediate postoperative radiographs demonstrated satisfactory fracture reduction and appropriate implant positioning. However, during routine follow-up visits, the patient reported persistent pain and difficulty with ambulation. At six months, the X-rays show malunion, and the patient was treated conservatively with vitamin D supplementation, pulsed electromagnetic field (PEMF) therapy, and rehabilitation.

Implant fatigue failure and salvage procedure

**Figure 1:**
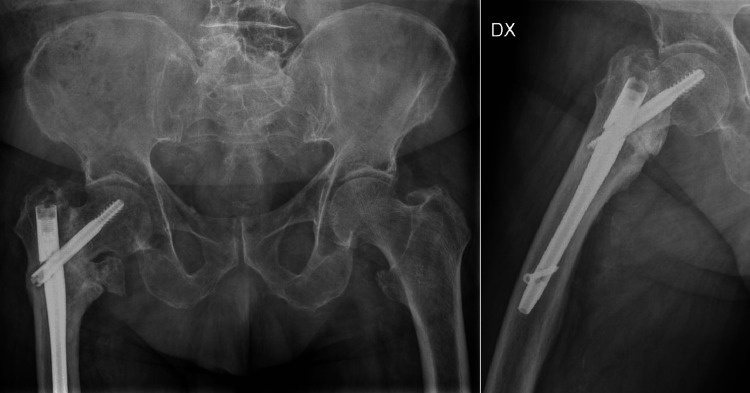
Plain antero-posterior radiographs showing nonunion of a pertrochanteric fracture with fatigue failure and breakage of the Gamma cephalomedullary nail

**Figure 2:**
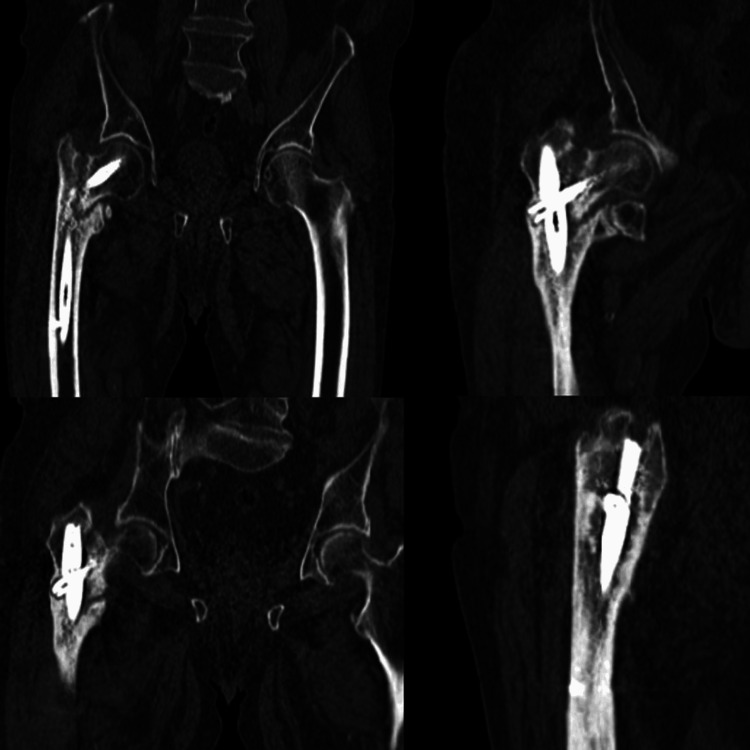
Computed tomography scan demonstrating nonunion of a pertrochanteric fracture with fatigue failure and breakage of the Gamma cephalomedullary nail

At the 13-month follow-up, the patient reported progressive hip pain, intolerance to weight-bearing, and perceived limb shortening. Radiographic evaluation (Figure 1) and computed tomography (Figure 2) demonstrated a nonunion of the pertrochanteric fracture associated with implant fatigue failure, showing complete breakage of the Gamma nail in the isthmic region.

The patient was completely independent prior to the injuries and had a normal clinical condition of the hip before the fracture. Considering the patient’s advanced age, functional demands, and the coexistence of hip osteoarthritis, conversion to THA was deemed the most appropriate treatment option. Despite preoperative concerns regarding intraoperative blood loss, the patient’s refusal of allogeneic blood transfusion necessitated meticulous planning of perioperative blood conservation measures. Preoperative laboratory investigations ruled out infection, showing normal white blood cell, ESR, and CRP values, and revealed a hemoglobin level of 14.1 g/dL (range 13-17).

Surgical procedure

After obtaining informed consent, the operation was performed under regional anesthesia to minimize hemodynamic fluctuations. The patient was positioned in the lateral decubitus position, and a direct lateral (Hardinge) approach was used. The surgical steps included proximal femoral resection and removal of the cephalic lag screw and the fractured Gamma nail, followed by reconstruction with a cementless total hip prosthesis.

The intraoperative blood conservation strategies included autologous cell salvage, the intravenous administration of tranexamic acid (TXA) to reduce blood loss, and meticulous surgical hemostasis throughout the procedure.

Postoperative management and early rehabilitation

The patient remained hemodynamically stable throughout the perioperative period, with hemoglobin levels consistently above 8 g/dL (range 13-17). Early mobilization was initiated on the second postoperative day using two crutches, with gradual progression to full weight-bearing, as tolerated.

Long-term clinical and radiographic outcomes

**Figure 3:**
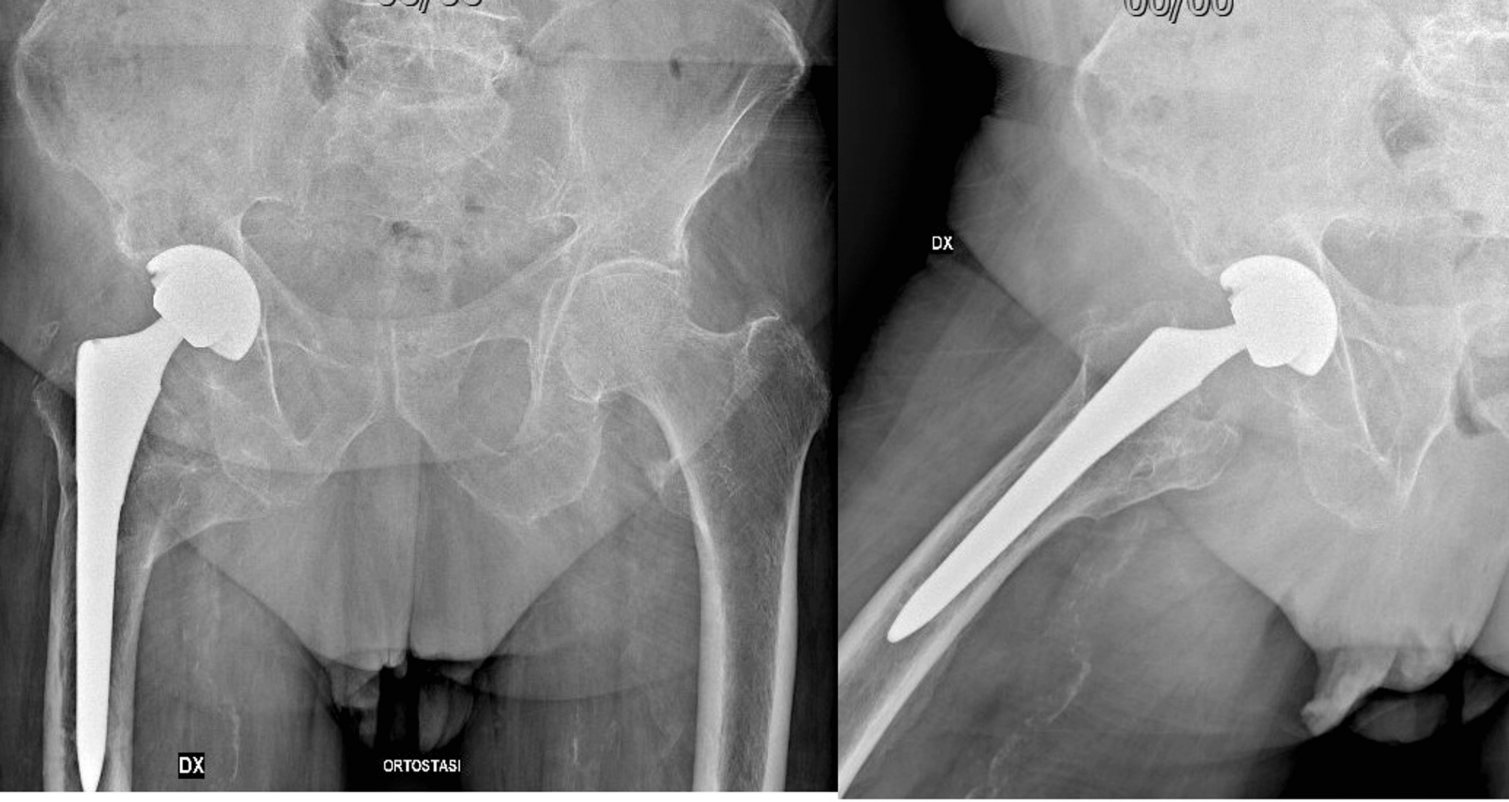
Anteroposterior radiographs of the pelvis and operated hip at the 10-year follow-up, showing stable cementless total hip arthroplasty with no evidence of loosening

Postoperative one, two, and six-month follow-ups showed good functional recovery of the patient, and the X-ray at one month showed good positioning of the prosthesis. At the one-year follow-up, the patient demonstrated painless ambulation and radiographic evidence of a well-fixed implant without signs of loosening or migration. At the 10-year follow-up, clinical evaluation confirmed excellent functional recovery, with a Harris Hip Score of 89 points (indicating good function) and satisfactory radiographic evolution showing stable component fixation and preserved bone stock (Figure 3).

No postoperative complications, such as infection, dislocation, or periprosthetic fracture, were observed during the entire follow-up period.

## Discussion

Gamma cephalomedullary nails are widely indicated for internal fixation of pertrochanteric and pathological fractures, as well as a revision option in selected cases of fixation failure [11-13]. One of the primary causes of Gamma nail failure is nonunion, which may lead to secondary fracture and subsequent implant breakage. This mechanical failure usually results from inadequate callus formation and persistent stress concentration at the nail, ultimately producing a fatigue fracture [14,15].

Several factors contribute to the development of nonunion and consequent implant failure, including inadequate initial fracture reduction, excessive displacement, suboptimal implant positioning, such as high or lateral placement of the cephalic lag screw, poor bone quality due to osteoporosis, and premature or excessive weight-bearing during the early postoperative period [16]. Gamma nail breakage secondary to nonunion is an exceptionally rare complication. In a retrospective cohort study conducted across tertiary public orthopedic hospitals in Western Australia, Lambers et al. reported cephalomedullary nail breakage in only 0.5% of cases [2].

When implant failure occurs, treatment selection depends on multiple factors, such as bone stock quality, in addition to patient comorbidities and functional demands. In younger patients with good bone quality, revision fixation with a long-stem intramedullary nail or plate osteosynthesis may be an appropriate option [12]. Conversely, in elderly patients with osteoporotic bone and coexisting hip osteoarthritis, conversion to THA is generally preferred, as it provides immediate pain relief, allows early mobilization, and ensures long-term functional recovery [13].

In the present case, considering the patient’s advanced age and the concomitant presence of hip osteoarthritis, conversion to cementless THA following removal of the broken nail was selected as the most suitable management strategy. Given the satisfactory intraoperative stability obtained during the surgical procedure, the decision was made to implant a cementless porous-coated stem without the need for a revision implant. One of the major concerns during such procedures, however, is perioperative blood loss, which may significantly affect postoperative outcomes, particularly in elderly or frail individuals. For patients who refuse allogeneic blood transfusion, this issue can become life-threatening [17]. Therefore, comprehensive surgical planning must include a multidisciplinary blood conservation protocol encompassing preoperative, intraoperative, and postoperative strategies. The most effective measures include preoperative hemoglobin optimization with iron supplementation and, when indicated, erythropoietin therapy, intraoperative hemostatic management with TXA and a meticulous surgical technique, and postoperative autologous blood reinfusion [18,19]. In our case, these combined strategies, including meticulous intraoperative hemostasis, proved effective in maintaining hemodynamic stability and preventing the need for allogeneic transfusion; as a result, a hematoma did not develop postoperatively, and hemoglobin levels were maintained within acceptable ranges, which facilitated the patient's recovery during the initial postoperative phase.

## Conclusions

Gamma nail breakage secondary to nonunion represents a rare but clinically significant complication in the management of pertrochanteric fractures. This event often reflects the cumulative effect of biological and mechanical factors, including impaired fracture healing, osteoporotic bone, and continuous cyclic loading on the implant. When fatigue failure occurs, careful preoperative evaluation and strategic surgical planning are mandatory to ensure optimal outcomes and to prevent further morbidity. In elderly patients with osteoporotic bone and concomitant degenerative hip disease, conversion to THA constitutes a reliable and definitive solution. This approach provides immediate pain relief, allows early mobilization, and leads to durable functional and radiographic recovery. In the present case, salvage THA performed after Gamma nail failure due to nonunion demonstrated excellent long-term results, confirming that this technique can effectively restore mobility and quality of life even after complex fixation failure.

This case underscores the importance of an individualized perioperative strategy, particularly in patients who decline an allogeneic blood transfusion. The implementation of a comprehensive blood management protocol, including preoperative hemoglobin optimization, intraoperative TXA administration, autologous cell salvage, and meticulous hemostasis, proved essential in maintaining hemodynamic stability and minimizing transfusion-related risk. Overall, this report reinforces the critical role of early detection of nonunion, accurate assessment of implant integrity, and timely surgical decision-making in preventing catastrophic implant failure. It also highlights that salvage total hip arthroplasty represents a consistent and effective treatment for cephalomedullary nail fatigue failure secondary to nonunion in pertrochanteric fractures, provided that meticulous preoperative planning and blood-conserving strategies are applied. The favorable 10-year outcome observed in this patient supports the long-term reliability of this approach in similar clinical scenarios.
